# Laparoscopy-assisted gastrectomy with D2 lymph node dissection for advanced gastric cancer without serosa invasion: a matched cohort study from South China

**DOI:** 10.1186/1477-7819-11-4

**Published:** 2013-01-11

**Authors:** Jian-Xian Lin, Chang-Ming Huang, Chao-Hui Zheng, Ping Li, Jian-Wei Xie, Jia-Bin Wang, Jun Lu

**Affiliations:** 1 Department of Gastric Surgery, Fujian Medical University Union Hospital, No 29 Xinquan Road, Fuzhou, Fujian Province, 350001, China

**Keywords:** Advanced gastric cancer, D2 lymphadenectomy, Laparoscopy-assisted gastrectomy, Matched cohort study, Open gastrectomy

## Abstract

**Background:**

Gastric cancer is a common malignancy worldwide and a common cause of death from cancer. Despite recent advances in multimodality treatment and targeted therapy, complete resection remains the only treatment that can lead to cure. This study was devised to investigate the technical feasibility, safety and oncologic efficacy of laparoscopy-assisted gastrectomy for advanced gastric cancer without serosa invasion.

**Methods:**

A retrospective matched cohort study was performed in south China comparing laparoscopy-assisted gastrectomy and open gastrectomy for advanced gastric cancer without serosa invasion. Eighty-three patients with advanced gastric cancer undergoing laparoscopy-assisted gastrectomy between January 2008 and December 2010 were enrolled. These patients were compared with 83 patients with advanced gastric cancer undergoing open gastrectomy during the same period.

**Results:**

There was no significant difference in clinicopathologic characteristics between the two groups. Regarding perioperative characteristics, the operation time and time to ground activities did not differ between the two groups, whereas the blood loss, transfused patient number, time to first flatus, time to resumption of diet, and postoperative hospital stay were significantly less in laparoscopy-assisted gastrectomy than in open gastrectomy (*P* <0.05). There was no statistically significant difference in postoperative morbidity and mortality. No significant difference in the number of lymph nodes dissected was observed between these two groups. There was no significant difference in the cumulative survival rate between the two groups.

**Conclusion:**

Laparoscopy-assisted gastrectomy with D2 lymphadenectomy is a safe and feasible procedure for advanced gastric cancer without serosa invasion. To be accepted as a choice treatment for advanced gastric cancer, well-designed randomized controlled trials comparing short-term and long-term outcomes between laparoscopy-assisted gastrectomy and open gastrectomy in a larger number of patients are necessary.

## Background

Gastric cancer, a common malignancy worldwide, is the second most common cause of death from cancer [1]. Despite recent advances in multimodality treatment and targeted therapy, complete resection remains the only treatment that can lead to cure. The use of laparoscopic techniques for early gastric cancer was first reported in 1994 [2] and, since then, many studies have reported benefits of the technique such as reduced blood loss, decreased pain, early recovery of bowel movements, and a shorter hospital stay [3-5]. Since 1999, when the first laparoscopy-assisted total gastrectomy with lymph node dissection for gastric cancer was reported [6], the use of laparoscopic gastrectomy for gastric cancer has been generally attempted in Japan and Korea, and the popularity of laparoscopic gastrectomy with lymph node dissection has increased rapidly. However, application of laparoscopic techniques for advanced gastric cancer (AGC) remains controversial because of the technical difficulty of extragastric lymphadenectomy and insufficient data related to the procedure’s oncologic adequacy. In the present study, we describe our experience with laparoscopy-assisted gastrectomy (LAG) in the treatment of AGC without serosa invasion, and evaluate the feasibility, safety and oncologic aspect of this approach through a matched cohort study.

## Methods

### Patients and materials

Between January 2008 and December 2010, 1,114 patients diagnosed with primary gastric cancer were treated with curative resection at the Department of Gastric Surgery, Fujian Medical University Union Hospital, Fuzhou, China. Of these patients, 632 underwent a laparoscopic approach and 482 underwent an open technique. Patients were informed of the possible complications of the procedure and the advantages and disadvantages of a laparoscopic compared with an open approach. Written informed consent was obtained from all patients prior to the operation.

The type of gastric resection was determined according to tumor location, size and depth of invasion. The D2 lymphadenectomies were undertaken according to the Japanese Gastric Cancer Society’s guidelines for the treatment of gastric cancer. Most nodal materials were separately dissected by the surgeons from the *en bloc* specimen at the end of the procedure, and the remaining nodes were identified and retrieved by specialized pathologists from formalin-fixed surgical specimens without using any specific technique to increase nodal retrieval rate. Paraffin-embedded nodes were stained with hematoxylin and eosin, and examined microscopically for metastases by specialized pathologists. Staging was done according to the seventh edition of the International Union Against Cancer tumor-node-metastasis classification [7].

Inclusion criteria were as follows: histologically confirmed adenocarcinoma of the stomach; performance status of Eastern Cooperative Oncology Group score 0 to 1; no evidence of distant metastasis or invasion to adjacent organs; and confinement without serosa invasion (pT2, pT3). Patients in the laparoscopic group were randomly matched to patients in the open group by age (±5 years), gender, gastrectomy extent and depth of invasion (pT2 and pT3) using a 1:1 interval matching method. All the patients received fluorouracil-based postoperative adjuvant chemotherapy for sixcycles, and no patients underwent preoperative chemotherapy. Follow-up was carried out by trained investigators through mail, telephone calls, visits to patients or records of the patients’ consultations at the outpatient clinic. Most patient routine follow-ups consisted of physical examination, laboratory tests (including carbohydrate antigen (CA)19-9, CA72-4 and carcinoembryonic antigenlevels), chest radiography, abdominopelvic ultrasonography or computed tomography, and an annual endoscopic examination. If gastrointestinal symptoms were reported, an additional examination was carried out. The survival time was from operation until the date that the survival information was collected or the date of death.

### Statistical analysis

Statistical analysis was performed using SPSS.v16.0 for Windows (SPSS Inc., Chicago, IL, USA). The statistical analysis was conducted by Student’s *t* test or chi-square test, and cumulative survival was compared by the Kaplan–Meier method and log rank test. Values of *P*<0.05 were considered statistically significant.

### Surgical procedures

The laparoscopy-assisted total gastrectomy surgical procedure is described here. The surgical techniques for lymph node dissection are principally the same in laparoscopy-assisted distal gastrectomy (LADG). Gastrointestinal continuity was restored in a Roux-en-Y fashion in total gastrectomy, and Billroth I or Billroth II reconstruction in distal gastrectomy.

All the patients were placed in a supine position with legs apart under general anesthesia. After the establishment of a pneumoperitoneum at 12 mmHg, one initial 10-mm trocar for a laparoscope was inserted below the umbilicus. The stomach and the peritoneal cavity were inspected to rule out adjacent organ invasion and peritoneal seeding using a 30° forward oblique laparoscope. A 10- to 12-mm port was inserted percutaneously in the left upper quadrant as a major hand port. A 5-mm trocar was placed at the contralateral site. Another two 5-mm trocars were respectively inserted in both the left and right lower quadrants. The surgeon stood on the left side of the patient, the assistant surgeon stood on the right, and the other surgeon handling the laparoscopy stood between the patient’s legs.

**Figure 1:**
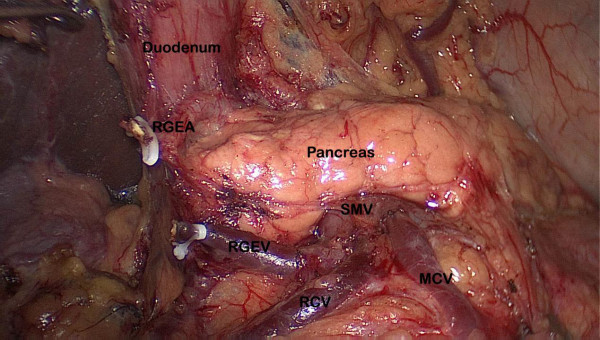
**Dissection of the lymph node numbers 14v and 6.** MCV: middle colic vein; RCV: right colic vein; REGV: right gastroepiploic vein; RGEA: right gastroepiploic artery; SMV: superior mesenteric vein.

**Figure 2:**
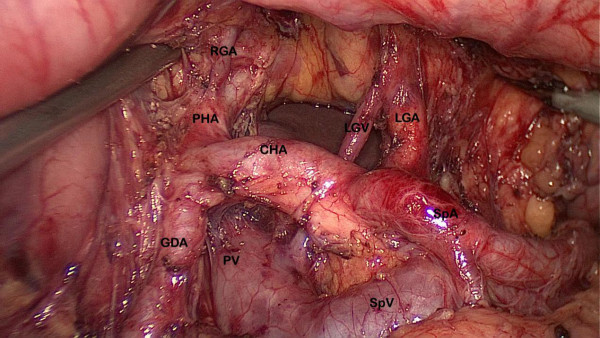
**Dissection of the lymph node numbers 7, 8, 9, 11p and 12a.** CHA: common hepatic artery; GDA: gastroduodenal artery; LGA: left gastric artery; LGV: left gastric vein; PHA: portal hepatic artery; PV: portal vein; SpA: splenic artery; SpV: splenic vein.

**Figure 3:**
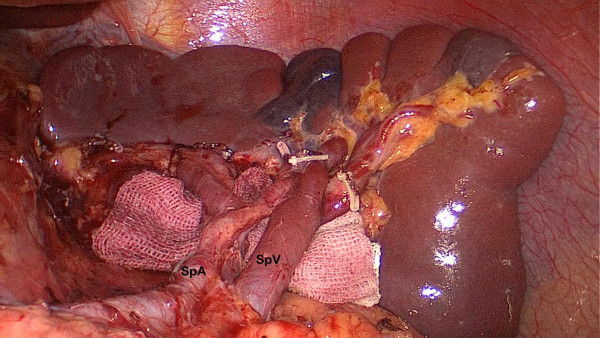
**Dissection of the lymph node numbers 11d and 10.** SpA: splenic artery; SpV: splenic vein.

The gastrocolic ligament was divided using an ultrasonically activated shear along the border of the transverse colon, thus including the greater omentum in the specimen to be resected. The dissection moved to the hepatic flexure and the pylorus. The right gastroepiploic vein was divided between titanium clips flush with the Henle’s trunk and ended up in the Fredet area, where group 14v was removed. The right gastroepiploic artery was vascularized and cut at its origin from the gastroduodenal artery with titanium clips, just above the pancreatic head, to dissect group 6 (Figure 1). The stomach was lifted headward to expose the gastropancreatic fold. The left gastric vein was carefully prepared and separately divided at the upper border of the pancreatic body and then the left gastric artery was vascularized to remove group 7. The lymph nodes along the proximal splenic artery (group 11p) were removed. Subsequently, the dissection was continued rightward along the artery to remove the nodes along the celiac axis and the common hepatic artery (group 9, 8a) by retraction on the left artery. The left gastric artery was cut between titanium clips at its origin from the celiac axis. The right gastric artery was divided at its origin from the common hepatic artery to dissect group 5. Along the border of the liver, the lesser omentum was dissected and the lymph nodes of the anterior region of the hepatoduodenal ligament (group 12a) were dissected and removed (Figure 2). Then, patients were tilted left-side-up about 20° to 30°and subjected to a 20° head-up tilt. The surgeon moved to stand between the patient’s legs; the assistant and the camera operator were both on the patient’s right side. The dissection of the gastrocolic ligament was continued toward the spleen with the removal of group 4sb; subsequently, the dissection was continued upward along the branches of splenic vessels to remove the nodes along the splenic vessels (group 10, 11d); all short gastric vessels (group 4sa) were divided close to the spleen (Figure 3). Before gastric transection, the cardiac nodes were dissected *en bloc* including right cardiac nodes (group1) and left cardiac nodes (group 2). The first part of the duodenum was dissected and then transected 2 cm below the pylorus with a 45-mm laparoscopic cartridge linear stapling device (endo-GIA, US Surgical Corporation, Norwalk, CT, USA) through a major hand port. Gastrectomy and anastomosis were extracorporeally performed using a handsewn method. The specimen was pulled out of the peritoneal cavity through the small laparotomy incision under the xiphoid (about 4 cm to 6 cm for distal gastrectomy and 6 cm to 8 cm for total gastrectomy).

For the open procedure, an approximately 15-cm to 20-cm incision was made from the falciform process to the periumbilical area. Distal gastrectomy and total gastrectomy with D2 lymph node dissection were performed basically.

All the operations were performed by one surgeon team with extensive experience both in open and laparoscopic gastric cancer surgery. For patients with gastric cancer located in the middle and upper third of the stomach, total gastrectomy was performed with Roux-en-Y reconstruction. In most patients with gastric cancer located in the lower third of the stomach, distal gastrectomy were performed with Billroth-I gastroduodenostomy. If the tumor had invaded the pylorus or duodenal ampulla, Billroth-II gastrojejunal anastomoses were produced. All the reconstructionswere performed with a circular stapler in an open manner.

## Results

### Clinicopathological characteristics of patients

Characteristics of the 166 case-matched patients (83 laparoscopic versus 83 open) are listed in Table 1. There were 142 men and 24 women, whose ages ranged from 28 to 85 years (61.3 ± 10.4 years). Both the LAG and open gastrectomy (OG) groups had 71 men and 12 women, 30 patients in pT2 and 53 patients in pT3, and 37 patients with total gastrectomy and 46 patients with distal gastrectomy. According to the International Union Against Cancer classification of gastric cancer [7], 32 patients (19.3%) were at stage Ib, 37 patients (22.3%) at stage IIa, 36 patients (21.7%) at stage IIb, 28 patients (16.9%) at stage IIIa, and 33 patients (19.9%) at stage IIIb. In the laparoscopic group, 24, 17 and 42 patients had their tumors located in the upper-, middle- and lower-third of the stomach, respectively, compared with 29, 11 and 43 patients in the open group. There was no statistically significant difference found in the majority of the demographic parameters between the two patient populations (Table 1).

**Table 1:** Clinicopathological characteristics

**Characteristics**	**Laparoscopy-assisted gastrectomy group (n = 83)**	**Open gastrectomy group (n = 83)**	***P***
Gender			1.000
Male	12	12	
Female	71	71	
Age(years)	61.6 ± 10.3	61.1 ± 10.5	0.777
Tumor diameter (cm)	4.6 ± 2.1	4.4 ± 2.2	0.631
Body mass index (kg/m^2^)	22.3	21.5	0.113
Tumor location			0.565
Upper	24	29	
Middle	17	11	
Lower	42	43	
Depth of invasion			1.000
T2	30	30	
T3	53	53	
pN stage			0.943
N0	30	29	
N1	17	20	
N2	17	15	
N3	19	19	
Tumor-node-metastasis stage			0.958
Ib	16	16	
IIa	19	18	
IIb	16	20	
IIIa	15	13	
IIIb	16	17	
Pathology			0.617
Differentiated	28	25	
Undifferentiated	55	58	
Gastrectomy extent			1.000
Total gastrectomy	37	37	
Distal gastrectomy	46	46	
Reconstruction			0.175
BillrothI	37	26	
BillrothII	6	10	
Roux-en-Y	40	47	

### Perioperative outcomes

There were no significant differences in volume of operation time (*P* = 0.214) and time to ground activities (*P* = 0.577) between the two groups. However, the blood loss (*P* <0.001), transfused patient number (*P* = 0.025), time of use of nonsteroidal anti-inflammatory drugs (*P* = 0.006), time to first flatus (*P* = 0.038), time to resumption of diet (*P* = 0.041), and postoperative hospital stay (*P* <0.001) were significantly less in the LAG group than in the OG group (Table 2).

**Table 2:** Perioperative results after laparoscopic and open gastrectomy

	**Laparoscopy-assisted gastrectomy group (n = 83)**	**Open gastrectomy group (n = 83)**	***P***
Operation time (min)	212.7 ± 57.2	226.4 ± 63.5	0.214
Blood loss (ml)	78.4 ± 77.9	200.4 ± 218.3	0.000
Transfused patients	3	11	0.025
Time of use of nonsteroidal anti-inflammatory drugs	3.1 ± 1.2	5.8 ± 2.0	0.006
Time to ground activities (days)	2.6 ± 1.1	2.7 ± 1.1	0.577
Time to first flatus (days)	2.9 ± 1.2	4.0 ± 1.0	0.038
Time to resumption of diet (days)	4.1 ± 1.5	5.5 ± 2.3	0.041
Postoperative hospital stay (days)	14.2 ± 7.2	17.2 ± 5.0	0.000

### Postoperative morbidity and mortality

The incidence of postoperative complications did not differ between the two groups (10 patients in the LAG group (12.0%) and 12 patients in the OG group (14.4%); *P* = 0.819). One patient in the LAG group died during their hospital stay because of complications arising from an anastomotic leak, diagnosed as septic shock, which developed into multiple organ disorder syndrome. Two patients in the OG group died during their hospital stay: one died of venous thromboembolism after surgery, the other died of sepsis induced by duodenal stump leakage (Table 3).

**Table 3:** Postoperative morbidities and mortalities

	**Laparoscopy-assisted gastrectomy group (n = 83)**	**Open gastrectomy group (n = 83)**	***P***
Complication	10	12	0.819
Duodenal stump leakage	0	1	
Anastomotic leakage	1	0	
Pancreatic fistula	1	1	
Lymphorrhea	1	1	
Intra-abdominal abscess	1	1	
Gastro-asthenia	2	2	
Anastomotic site bleeding	0	1	
Anastomotic straitly	1	1	
Venous thromboembolism	0	1	
Pulmonary infection	2	3	
Blood poisoning	1	0	
Postoperative mortality	1	2	1.000

### Lymph node retrieval in the laparoscopy-assisted and open gastrectomy groups

**Figure 4:**
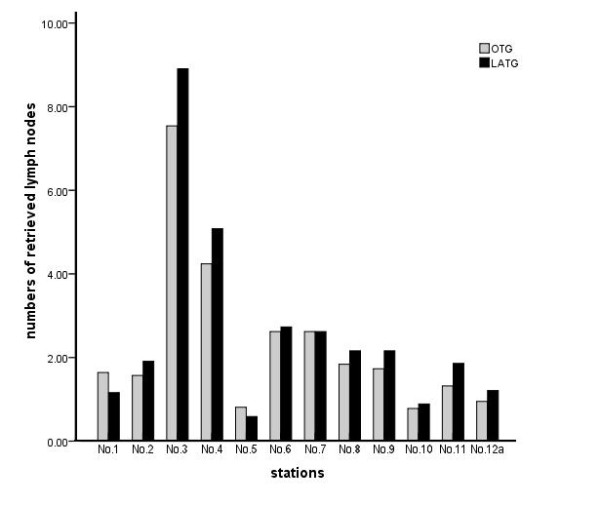
**The retrieved lymph nodes from laparoscopy-assisted total gastrectomy (black bar) and open total gastrectomy (gray bar).** There was no significant difference in the numbers of retrieved lymph nodes at each station.

**Figure 5:**
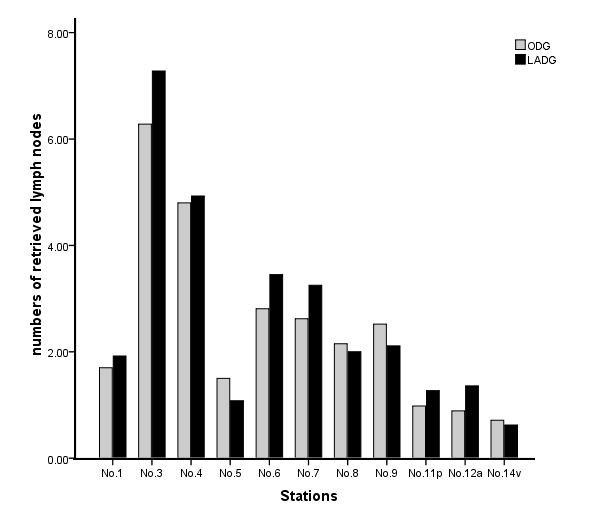
**The retrieved lymph nodes from laparoscopy-assisted distal gastrectomy (black bar) and open distal gastrectomy (gray bar).** There was no significant difference in the numbers of retrieved lymph nodes at each station.

The median of total lymph nodes was 28 per patient (range, 14 to 62; mean 29.1 ± 9.2). The total number of retrieved lymph nodes was not different between the two groups (30.2 ± 10.1 in the LAG group versus 28.0 ± 8.1 in the OG group; *P* = 0.103). No significant difference in the numbers of retrieved lymph nodes at each station was observed regardless of the gastrectomy extent (Figures 4 and 5).

### Survival after surgery

**Figure 6:**
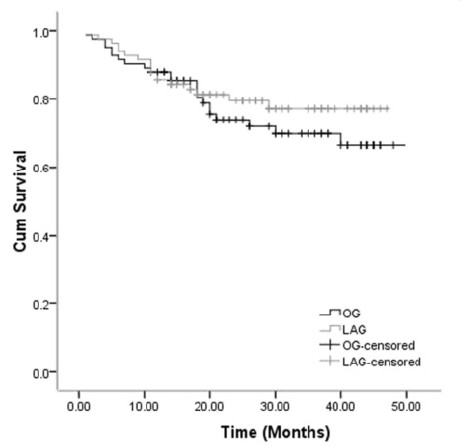
**Comparison of cumulative survival rate of laparoscopy-assisted gastrectomygroup and open gastrectomy group by log-rank test (*P*>0.05).**

The median follow-up for the entire cohort was 23.0 months (range, 12 to 50 months). The follow-up rate was 96.4%, with 160 patients involved (both LAG group and OG group had 80 patients). The overall 1-year survival rate was 88.0% in the LAG group and 85.5% in the OG group, and there was no significant difference in the survival curve between the two groups (Figure 6).

## Discussion

In the world literature, reports of laparoscopic techniques for treating patients with early gastric cancer have shown oncologic and long-term survival equivalency to the open technique, with known benefits to a minimally invasive approach [8,9]. For AGC, the Japanese Gastric Cancer Association has presented complete D2 lymphadenectomy including lymph nodes 10, 11p and 12a as the standard therapy. In fact, curative resection of AGC involves extended lymphadenectomy, which is well accepted by Eastern Asian countries, such as Japan, Korea and China, and some specialized centers in Europe, though it remains controversial worldwide [10-12]. Nevertheless, laparoscopic D2 lymph node dissection has not been widely investigated because it is considered to be technically difficult and was performed only in a few institutes by highly experienced surgeons [13-16]. In our hospital, D2 lymphadenectomy has been routinely performed.

An important consideration for new surgical procedures is the learning curve faced by those who will be performing them, as evidenced by experience from laparoscopic procedures and general surgical techniques [17-20]. The progress in the performance of a surgeon is delineated as the so-called learning curve [21]. Like other laparoscopic procedures, there is a learning curve associated with LAG, and many surgeons are starting to perform this treatment with the tacit acceptance of a lengthy operation time because they often perceive LAG as a complicated technique inevitably subject to the learning curve effect. A study by Kunisaki *et al*. [22] focusing on the surgical learning curve of LADG by a single surgeon showed the operating time was shortened to 230 min after 60 cases. Lee *et al*. [23] reviewed the cases of 257 patients who received distal gastrectomy (included 136LADGs and 120 ODGs); they found that the mean operation time was similar between the two groups. The first LAG for early gastric cancer was performed in April 2007 in our center. To ensure the quality of our surgical procedure, we undertook this study after ‘climbing up’ the learning curve, when we performed more than 50 cases. In the present study, there were no significant differences in operation time for LAG and OG groups (212.7 ± 57.2 min versus 226.4 ± 63.5 min, *P* = 0.214). However, we found that patients undergoing laparoscopy-assisted surgery had better postoperative recovery, with less blood loss, quicker intestinal functional recovery, and shorter hospital stay than those undergoing conventional open surgery, similar to many reported studies [24-26]. In addition, the safety of laparoscopic gastrectomy is very important for LAG. Kitano *et al*. [27] reported a morbidity of 14.8% in a multicenter trial with 1,294 patients undergoing laparoscopic gastrectomy. The KLASS trial [28], a Korean multicenter prospective randomized trial that included 179 patients undergoing laparoscopy-assisted and 163 patients undergoing open distal gastrectomy, reported an 11.6% early morbidity for the LAG group and 15.1% for the OG group, with a mortality of 1% for the LAG group. However,in this study there was no difference in the incidence of morbidity between the LAG and OG groups (12.0% versus 14.4%, *P* = 0.819). Therefore, LAG for gastric cancer may be acceptable from this viewpoint. All of the above suggest that LAG with D2 lymph node dissection for AGC without serosa invasion is a safe and feasible choice.

Nowadays, more and more studies show that the procedure for gastrectomy with complete D2 lymph node dissection is well established and accepted as a standard practice for the treatment of AGC. So, besides the technical feasibility and favorable clinical outcomes of LAG, the quality of lymphadenectomy is the most important factor in performing LAG with D2 dissection. A Japanese study found that adequate staging was possible for 86% of the patients who underwent LADG with D2 dissection because more than 15 lymph nodes, the minimum requirement for tumor-node-metastasis staging, were retrieved. However, the total number of lymph nodes and the nodes at station 4, 6, 9 and 11 were greater in the ODG group than in the LADG group [29]. A similar study conducted by Huscher *et al*. [30] showed that there was no significant difference in the number of retrieved lymph nodes at each station. Song *et al*. [31] enrolled 75 patients who received standard D2 lymph nodes dissection (44 underwent LADG, and 31 underwent ODG), and found no significant differences in the total number of retrieved lymph nodes or node stations between the two groups. They suggested that LADG with D2 lymph node dissection is oncologically compatible with OG. In the current study, as a way of comparing the oncologic aspect of quality control between the LAG and OG groups, we compared the total number of retrieved lymph nodes and numbers of nodes by their stations. The result showed that no significant difference in the number of retrieved lymph nodes or nodes at each station was observed regardless of the gastrectomy extent. It is proved that LAG with D2 lymph node dissection is technically possible, and the number of retrieved lymph node was sufficient for accurate staging.

The long-term oncologic result is very important in the use of laparoscopic gastrectomy. Although few studies on the outcome of LAG for AGC with T2 and T3 depth of invasion have been published previously, the results of the present study and other reports [32-34] show good outcomes. Shuang *et al*. [32] compared 35 patients undergoing LAG with 35 matched OG, and their results indicated technical feasibility and equivalent short-term recurrence-free survival of laparoscopic gastrectomy for gastric cancer when compared with the open procedure. In this series, the survival rates after LAG were excellent. The 1-year survival rate after LAG was 88.0%, similar to the OG group, and there was no significant difference in the survival curve between the two groups.

## Conclusions

Although the present study was not a randomized controlled study and the follow-up period was not long enough, the survival rate of patients with AGC who underwent LAG was shown to be good. This study has shown that LAG for AGC has several advantages over OG, and LAG yielded similar oncologic outcomes including complication rates and cumulative survival after 50 months of follow-up. To be accepted as a choice treatment for AGC, it is necessary to conduct a well-designed prospective trial to assess long-term outcomes.

## Abbreviations

AGC: Advanced gastric cancer; CA: Carbohydrate antigen; LADG: Laparoscopy-assisted distal gastrectomy; LAG: Laparoscopy-assisted gastrectomy; OG: Open gastrectomy.

## Competing interests

The authors declare that they have no competing interests.

## Authors’ contributions

JX Lin and CM Huang conceived of the study, analyzed the data and drafted the manuscript; CH Zheng helped revise the manuscript critically for important intellectual content; P Li, JW Xie, JB Wang and J Lu helped collect data and design the study. All authors read and approved the final manuscript.
